# Study of Middleware for Internet of Healthcare Things and Their Applications

**DOI:** 10.1007/978-3-030-51517-1_18

**Published:** 2020-05-31

**Authors:** Ghofrane Fersi

**Affiliations:** 8 Digital Research Centre of Sfax, Sfax, Tunisia; 9 Institut Mines-Télécom, CNRS, Paris, France; 10 Université de Sherbrooke, Sherbrooke, QC Canada; 11 Digital Research Centre of Sfax, Sfax, Tunisia; 12 University of Sfax, Sfax, Tunisia; 13 Laboratory of Development and Control of Distributed Applications (ReDCAD), University of Sfax, Sfax, Tunisia; 14 Higher Institute of Applied Sciences and Technology of Sousse (ISSAT), University of Sousse, Sousse, Tunisia

**Keywords:** Middleware, Healthcare, Internet of Healthcare Things

## Abstract

The rapid proliferation and miniaturization of the wireless and embedded devices has led to the invasion of the Internet of Things in many domains and has reached the healthcare sector to form what is called the Internet of Healthcare Things (IoHT) . The growing number of the applications in the Internet of Healthcare Things as well as the overwhelming number of heterogeneous medical devices that should interact in this network has put the researchers and developers in front of lot of challenges: How to facilitate the implementation of the various healthcare applications? And how to ease the integration of new devices and make their interoperation a transparent task for the developers? To fulfill these requirements, lot of middleware have been proposed. In this paper we provide a complete study on the existing middleware for IoHT and specify their applications, we propose a taxonomy for them and we present their main advantages and drawbacks.

## Introduction

The interest to the healthcare digitalization is growing during these last years due to its multiple benefits [1]. Effectively, there is a growing need to monitor the patients behavior and/or status and notify the doctors and the relatives of any danger according the patient’s health. Such tight monitoring cannot be ensured by nurses or caregivers. Internet of Things (IoT) represents here a suitable and efficient solution for this [2]. Effectively, IoT is made up of tiny devices that can easily communicate and cooperate together to achieve a common task. This is exactly what is needed in healthcare: tiny devices that might be portable (off the body), wearable (on the body) or implantable (in the body), track the patient’s status. The application of IoT in the healthcare is called Internet of Healthcare Things. This new concept has certainly improved the healthcare services and increased its efficiency. However, it is facing lot of challenges. Effectively, healthcare applications are compelled to deal with medical devices having different protocols, various capabilities and working with different standards. These devices are also mobile and prone to failure. So it is important to conceive a layer between the physical devices and the application layer that hides all these diversities and deals with these challenges transparently to the end devices. This layer is called middleware [15]. We study in this paper the various middleware that have been proposed for IoHT and discuss them.

Our paper is organized as follows: We present in the second section a study of the existing middleware for IoHT. In the third section, we discuss the proposed solutions. And finally, section four concludes the paper.

## Existing Middleware for IoHT and Their Corresponding Applications

We present in this section the most important middleware that have been proposed during these last years for the IoHT. We propose to classify them according to their main technical directives.

### Fog-Based Middleware

Fog computing is considered by many researches as a middleware between the IoT and the cloud computing. This intermediate layer [3] reduces drastically the latency and affords the required processing and storage needs to the healthcare IoT devices. Effectively, many healthcare situations require a timely reaction. A IoT security middleware for use between IoT, fog and cloud computing, has been proposed in [4]. The main aim of this middleware is to ensure security and better network performance during churns. ‘Session resumption’ algorithm has been used by the authors to help a recently disconnected node to regain its encrypted session. This middleware can be applied in rural healthcare and public safety applications. In [5], fog nodes that are placed near to the patient’s location will host a privacy middleware that ensures the user’s privacy. In these nodes, data are stocked in clear and are accessed rapidly. Authors in [6] argue that using fog-based middleware in healthcare ensures the agregation of data that are generated from the patients with ensuring their privacy and confidentiality. Hence, this will lighten the burden of security processing in IoT healthcare devices.

### Publish/Subscribe-Based Middleware

The multiplicity of events in the healthcare domain favors the use of Publish/Subscribe middleware due to their ability to deliver asynchronously multiple events from their sources to their subscribers. In [7], an IoT middleware has been proposed to help people with special needs. In this middleware, there is only one powerful component (NCeH) that will be deployed to every node that belongs to the network, in order to have a uniform configuration. This component is made up of various modules that ensure the communication between it and the other nodes and also to enable it to self-configure itself based on remote commands, and to analyze the received data so that the appropriate decision can be taken.

The proposed middleware [8] is used to classify the patients movements and count their steps number. The required materials are 2 sensors with triaxial accelerometer that should be placed on the knee and on belt, and a PDA or a smart phone to acquire the collected data by the sensors and process the classification. VIRTUS uses XMPP to know if the message is delivered to the destination or not. Also, the use of XMPP allows to verify if the destination is not connected when the message is received or not. This middleware is adaptable to modules composition changes without the need to restart the system.

### Web of Things-Based Middleware

Authors in [20] and [10] argue that Web of Thing-based middleware are suitable to ensure interoperability since all the devices can be abstracted as a web resource.The ECOHelath middleware proposed in [10], connects doctors and patients to ease the health monitoring system and offers more accurate patients diagnosis. This middleware is based on the Web of Things (WoT) paradigm in which the physical devices are digitalized to ensure the efficient use of their data in different applications. In this middleware, there are body sensors that send their related data through a web interface that is provided by a Visualization and Management module. This interface helps doctors to track their patients’ status. All the medical data are stored by the storage module in a relational database with the possible support of cloud computing. The proposed middleware has been applied to monitor the patients’ heartbeats and blood pressure. ECG sensors are used to measure the heartbeats and discover heart pathologies. Blood pressure oscillometer sensor measures the arterial pressure. $\upmu$WoTOP (micro Web of Things Open Platform) [11] has the ability to integrate heterogeneous biometric sensors. The data collection and notifications transmission are ensured by gateways. This middleware relies on Web technologies such as Html, REST. Its web of things strategy offered it the ability to be suitable at the same time for many healthcare applications like: Fall detection for elderlies, faint detection, abnormal behavior detection and freezing detector for Parkinson patients.

The main aim of the Sphere [12] middleware is to offer a flexible platform to integrate different sensors types and ensure their cooperation to achieve a given objective such as fall detection, Activity of Daily Living (ADL) recognition, and behavior anomaly detection.

### SOA Based Middleware

SOA-based middleware rely mainly on services that offer a public functionnality through an interface while hiding their internal details. The main advantage of these middleware is their ability to reuse multiple devices with ensuring their communication as providers and requestors independently from any underlying architecture. Linksmart [14] is a SOA-based middleware that has proved its ability to interoperate with various devices. This middleware is widely used in healthcare to monitor patients. The Smart Homes for All (SM4All) middleware framework [13] has been proposed to help people with special needs in their homes. This middleware integrates multiple protocols such as UPnP into the OSGi framework and is able to interoperate with devices employing Zigbee, Bluetooth. This allows heterogeneous devices to connect dynamically and interact with each other in person-centric surrounding.

The main objective of Uranus middleware [16] is to afford Ambient Assisted Living (AAL) for users with vital signs monitoring. This ensures a rapid prototyping for multiple applications working on healthcare and users wellness. Authors presented two case studies that have been tested using Uranus middleware. In the first, the oxygen level in the blood of a chronically ill patient is monitored at his home. To fulfill the requirements of this case study, an oximeter should be attached to the patient to measure the oxygen level and send it to a smart phone which transmits it to the doctor. The second case study aims to monitor patients that should be injected with radioactive substance. This monitoring alerts nurses in the case of patient’s complications after injection and also supervise the radiation level in order to specify the convenient examination time (Each examination type requires a specific radiation level to deliver the accurate results). To achieve this, each patient is equipped with an RFID tag, a PDA and an ECG sensor. These equipments in addition to the service discovery ensured by the middleware enable to monitor the patient’s heart beats. The patient location is updated when he moves from a room to another in order to track his status (still waiting, in the examination state, injected and awaiting that the radiation level reduces). These events are traduced using semantic information.

The contribution [9] is mainly used in sleep monitoring and bedsore prevention. The patient’s positions in the bed are specified and classified according to the collected RSSI of the sensors using SVM classification method. These positions give an idea about the patient’s sleep and can prevent from bedsore risks. This middleware helps the caregivers and eases their job by keeping track of the patient’s position in the bed and decides when and how the patient should change his position to avoid bedsores. It is made up of two layers. The middleware [17], is able to monitor and offer assistance to disabled people. The system functionalities are dispatched and divided into independent services. An ECG sensor monitors the heart activity.

### Event-Driven Middleware

In the event-driven middleware, all the middleware functionnalities are based on events going from events production to the reaction to events. Authors in [18] consider that event driven middleware is suitable to the context of healthcare, since the sensors reading according to the patient’s status and/or activity change over the time. Also, the majority of medical devices work according to the event driven process. For example, when the heartbeats rate exceeds a predefined value, a notification is triggered. Furthermore, the event driven process reinforces the data abstraction that is needed to ensure applications interoperability. The proposed middleware is dedicated for smartphone like devices that are compelled to interact with a changing set of wireless nodes in a medical environment. The proposed middleware has the ability to multiplex the received data from the various sensors in order to fit multiple applications simultaneously.

The contribution has been applied in three applications. The first is the fitness support in which the user’s physical activity is tracked by dedicated sensors: a wearable accelerometer to track the user’s status (walking, sitting, standing etc.) and a chest strap and two embedded sensors in the weightlifting gloves to figure out the user’s body exercise. This application motivates the user and helps him to track his sports activities. The second application is the telemonitoring. The main objective of this application is to monitor remotely the patient’s status. To this end, the middleware collects information about the patient’s weight, pressure, heart rate, ECG, etc. When the heart rate of the patient is not suitable (abnormal) to his activities, an alarm is sent. The third application is elderly care that monitors the elderly daily activities and reminds him whenever he forgets an important task.

### Message Oriented Middleware (MoM)

Message oriented middleware have the ability to exchange important number of messages between distributed applications. A MoM in [21] is proposed to unify the access to all the medical devices in order to ease the data collection from the patients to the doctors. To this end, “Advanced Message Queuing Protocol (AMQP)” is employed to ensure data transfer according predefined standards with the required interoperability. To increase the security of the middleware, a RESTful application has been added to verify which manager is allowed to access which data.

### Real Time Publish Subscribe Based Middleware

The Real-Time Publish Subscribe (RTPS) middleware is well suited for the real-time distributed applications. That’s why authors in [19] decided to port this middleware to healthcare. In this middleware, each medical device such as temperature probe, Capnometer, ElectroCardiogram, has the function of a publisher. It collects and publishes the patient’s measurements via fast Ethernet LAN. The published information is sent to the corresponding subscribers.

**Table 1.** Taxonomy of middleware for IoHT

Healthcare domain	Contribution	Required devices	Middleware class
Patients privacy preservation	[3–6]	NM	Fog-based
Fall/Faint detection	[12]	Wearable sensors with accelerometers	
	[11]	Accelerometer, blood pressure wristband	Web of Things
	[17]	Air pressure sensor, triaxial accelerometer	SOA
Freezing detection	[11]	Accelerometer	Web of Things
Patients monitoring	[13, 14]	NM	SOA
	[18]	Wearable accelerometer, chest strap	Event-driven
	[10]	ECG sensors, blood Pressure oscillometer, Cooking health sensor, biometric chest belt	Web of Things
	[11]	Blood pressure, accelerometer, Mood ring	Web of Things
	[8]	Sensors, accelerometer	Publish/Subscribe
AAL+ADL	[16]	ECG sensor, Oximeter, Zigbee, THL Sensor	SOA
	[9]	NM	
	[12]	Environmental sensors, video sensors, RGB-D sensors	Web of Things
Bedsore prevention	[9]	Bed pressure pad, wireless PIR detectors, luminance sensors	SOA
Radio activity injection monitoring	[16]	RFID tag, ECG sensor	SOA

## Discussion

As we have stated in the previous sections, the middleware for healthcare of things have covered lot of healthcare domains. Table 1 gives an overview of these middleware, their applications and their required medical devices (NM means not mentioned in the related paper). We notice that a same application can be ensured in many cases by different middleware belonging to different classes. For example, patients monitoring applications are implemented using SOA-based or Event-driven or Web of things or Publish/subscribe middleware. There are also some middleware solutions like [11] and [9] that offered a very flexible architecture that allowed them to be applied in a wide applications range. So, we should now answer at this crucial question: What is the most suitable middleware for Internet of healthcare things?

The answer at this question is not trivial. Effectively, we see that there is no ‘perfect’ solution since there are miscillaneous applications in the healthcare that have different uses cases with different constraints. For example, in some healthcare applications, there are lot of events that occur and require a quick reaction, such the case of an application that monitors the vital signs of a patient in a reanimation unit. Any modification in these signs (heart beats rate decrease, respiration problem, etc.) should trigger an event that must be handled rapidly. In these situations, an event-based middleware is a suitable candidate.

In SOA, there are service providers, service consumers and service registry. The service consumers search their required service from a service registry. They inform the corresponding service provider if the service is found. This architecture is well-suited for heterogeneous devices. And it ensures efficient service composition. However it does not fit real time applications. Many health scenario rely on one to many communication like the dissemination of a relevant healthcare information to a group of patients. In these situations, Publish/Subscribe is more suitable than the request/response strategy because it offers lower delays. But, if many situations are present simultaneously, how to choose the best middleware?

The answer is: Why to choose if we can combine? Effectively, most of these middleware classes are not mutually exclusive! We can conceive a middleware that is at the same time an event-based and a Web of Things-based for example. A middleware can even contain multiple components and each component can be based on different middleware class.

Based on the studied middleware, we can confirm that there are up to now lot of challenges that are not overcame. Indeed, the majority of the proposed middleware are conceived to a specific application and are not able to be adaptable to other applications without applying core modifications. Also, interoperability is still a crucial issue due to the continuous emergence of new medical devices with new technologies and new standards. Furthermore, the progress of the security middleware is countered by the progress of the security attacks, which lets security one of the most leading open issues that need more investigation.

## Conclusion

We studied in this paper existing middleware for the Internet of Healthcare Things and specified their various applications. This study conducted us to conclude that in spite of the diversity of the proposed middleware, none of them has the ability to fulfill all the healthcare requirements. This urges to the openess and the collaboration between these middleware to have a more robust middleware verifying the trade off between the IoHT needs and devices technical limits.
